# Complex Systems Modeling for Obesity Research

**Published:** 2009-06-15

**Authors:** Ross A. Hammond

**Affiliations:** Center on Social and Economic Dynamics, Economic Studies Program, The Brookings Institution

## Abstract

The obesity epidemic has grown rapidly into a major public health challenge, in the United States and worldwide. The scope and scale of the obesity epidemic motivate an urgent need for well-crafted policy interventions to prevent further spread and (potentially) to reverse the epidemic. Yet several attributes of the epidemic make it an especially challenging problem both to study and to combat. This article shows that these attributes — the great breadth in levels of scale involved, the substantial diversity of relevant actors, and the multiplicity of mechanisms implicated — are characteristic of a complex adaptive system. It argues that the obesity epidemic is driven by such a system and that lessons and techniques from the field of complexity science can help inform both scientific study of obesity and effective policies to combat obesity. The article gives an overview of modeling techniques especially well suited to study the rich and complex dynamics of obesity and to inform policy design.

## The Obesity Epidemic as a Complex System

The obesity epidemic has grown rapidly over the last few decades into a major public health challenge in the United States and, increasingly, worldwide. Between 1970 and 2000, the percentage of obese Americans doubled to almost 30% (1), with two-thirds of Americans now overweight (2). Similar obesity epidemics are under way across the globe (3-8). Worldwide, nearly half a billion were overweight or obese in 2002 (9).

The growth of the obesity epidemic has significant implications for public health (10) and health care costs (11). Obesity in children is also growing rapidly (9,12), presenting immediate health risks and suggesting the potential for even larger future increases in adult obesity unless the epidemic is contained. One public health researcher argues that obesity may become "the gravest and most poorly controlled public health threat of our time" (13). Both the scope and the scale of the obesity epidemic motivate an urgent need for well-crafted interventions to prevent further spread and to (potentially) lower current rates of overweight and obesity.

Yet 3 attributes of the obesity epidemic make it an especially challenging problem — both to study and to combat. First is the huge range in the levels of scale involved (14). Empirical evidence suggests important (and potentially interconnected) effects at levels including genes (15-18), neurobiology (19-22), psychology (23-28), family structure and influences (29-32), social context and social norms (33-45), environment (46,47), markets (11,48,49), and public policy (50,51). Not only do these levels entail very different pathways of effect and diverse methodologies for measurement, they are also usually the province of very different fields of science (from genetics to neuroscience to economics and political science).

A second challenging characteristic is the diversity of actors who potentially affect individual energy balance (and thus population levels of obesity). These might include families, schools, retailers, industry, government agencies, the media, health care providers, city planners, architects, employers, insurance companies, and many others. Each of these actors has different goals, motivations, constraints, sources of information, modes of decision making, and types of connection to other actors. Interventions may affect each differently, and each has a different sphere of potential influence as an agent of change. Interventions that do not take into account the diversity of these actors cannot leverage potential synergies. They also run the risk that successful interventions in one area may be counteracted by responses of other actors.

A third challenge is the multiplicity of mechanisms at work in the obesity epidemic. For example, the role of dopamine-mediated reward and the mesocorticolimbic pathway in eating is well documented (52,53). Genes such as the dopamine-4 (*DRD4*) and dopamine-2 (*DRD2*) receptors can influence the dopamine system and affect feeding and reward (15-17). Individual choices about food are also influenced by neurobiological systems, such as executive control and the dopamine-striatal system (19-22), and by measurable psychological factors such as dietary disinhibition (23-25) and sensitivity to reward (26-28). Early childhood and prenatal family influence can play a strong role in subsequent obesity through several mechanisms (29-32). Social norms and social contextual influences affect food consumption directly (33-36) and indirectly via body image (39-43) and social capital (44,45). Obesity is known to spread through social networks (37-38) by an as-yet-unidentified mechanism. Prices can strongly influence food choice (11,48,49), as can built environment (46,47).

Even where mechanisms of effect are clear, the *linkages and feedback between these mechanisms* are not well studied or well understood. Furthermore, no single mechanism appears able to account for all that we know about the obesity epidemic. For example, markets and prices provide a compelling explanation of the overall upward trend in obesity rates but do not explain the important disparities in incidence by sociodemographic groups (54,55), nor provide insight into why obesity appears to move through social networks (37-38). Neurobiological and genetic mechanisms help explain the resilience of obesity at both the social and individual levels but have difficulty explaining the timing and speed of the epidemic and its spatial clustering. Environmental explanations capture the spatial and demographic variability in obesity incidence but cannot explain its apparent spread across longer distances through networks or its variation within spatially coherent demographic groups.

In sum, the obesity epidemic is a particularly challenging problem because it results from a system containing a diverse set of actors, at many different levels of scale, with differing individual motivations and priorities. This system has many moving parts and operative pathways, which interact to produce rich variation in outcomes that cannot be reduced to a single mechanism. Taken together, these features are classic characteristics of a *complex adaptive system* (CAS).

A CAS is one composed of many heterogeneous pieces, interacting with each other in subtle or nonlinear ways that strongly influence the overall behavior of the system. The CAS perspective has proved enlightening in the study of economic, political, social, physical, and biological systems (56-58). CASs share many general properties, including:

- *Individuality*: CASs are often multilevel but are usually driven by decentralized, local interaction of constituent parts. Each level is composed of autonomous actors who adapt their behavior individually. Actors can be people but also larger social units such as firms and governments, and smaller biological units such as cells and genes.
- *Heterogeneity*: Substantial diversity among actors at each level — in goals, rules, adaptive repertoire, and constraints — can shape dynamics of a CAS in important ways.
- *Interdependence*: CASs usually contain many interdependent interacting pieces, connected across different levels. System dynamics are often characterized by feedback and substantial nonlinearity.
- *Emergence:* CASs are often characterized by emergent, unexpected phenomena — patterns of collective behavior that form in the system are difficult to predict from separate understanding of each individual element.
- *Tipping*: CASs are also often characterized by "tipping." Nonlinearity means that the impacts caused by small changes can seem hugely out of proportion. The system may spend long periods in a state of relative stability, yet be easily "tipped" to another state by a disturbance that pushes it across a threshold.

These characteristics make the study and management of complex systems especially challenging. Valuable insights about such systems, along with strategies for intervention, can be gained from the relatively new, interdisciplinary field of complexity science.

## Implications for Science and Policy Design

The complexity of the systems underlying the obesity epidemic has important implications for scientific study of obesity, for policy and the design of interventions aimed at changing the course of the obesity epidemic, and for modeling to facilitate both of these goals.

### Scientific study

Linkages and feedback between mechanisms (and between levels of analysis) are often important determinants of dynamics in complex systems. In the case of obesity, these links are not well understood, although many individual mechanisms operating at a single level have been identified. Because no one mechanism appears able to completely explain all important aspects of the obesity epidemic (its timing, scope, variance, distribution, etc), greater understanding may require approaches that combine mechanisms and explore their interplay. This means that division of scientific study by traditional disciplinary boundaries may be hampering a full understanding of the problem — new insight can come from *cross-disciplinary* approaches. Similarly, methods and approaches that are systems oriented and *multilevel* in scope are needed (14,59,60) to capture linkages between mechanisms at different levels. See below for discussion of suitable methods.

### Policy and intervention design

Complexity can be a significant challenge for policy makers and for the design of interventions. The interconnected dynamics of a complex system may lead policy design to overlook potential synergies, and successful interventions in a single area may be counteracted by responses elsewhere in the system. Policies that do not take into account the full set of actors and their responses can even backfire dramatically, as illustrated by the Lake Victoria catastrophe (61,62). In 1960, a nonnative species of fish (the Nile perch) was introduced into Lake Victoria, with the policy goal of improving the health and wealth of the communities of people surrounding the lake in Kenya, Tanzania, and Uganda through this new source of protein. But the policy did not take into account the other actors in the system — specifically, the other organisms that formed the complex ecosystems of the lake. Although the perch initially appeared to be a success, its introduction into the lake set off a chain reaction in the lake ecosystem. The perch wiped out the native cichlid species of fish, which were crucial in controlling a species of snail living in the lake. The snails flourished, and with them the larvae of schistosomes, to whom they play host. Schistosomes are the cause of the often-fatal disease of bilharzia in humans, and their exploding numbers created a public health and economic crisis. Thus, the original policy goal (improving the health of the surrounding communities of humans) backfired because the reaction of another set of actors in the system was not anticipated. Efforts to reduce obesity might face similar difficulties if systemic diversity is not factored into policy design.

Other characteristics of complex systems pose challenges for policy design as well. Nonlinearity makes prediction difficult — multiple forces shape the future, and their effects do not aggregate simply. Heterogeneity means that any given intervention may not work equally well across all contexts or subgroups. Decentralized dynamics can be a challenge because many conventional policy levers and intervention implementations are centralized or "top-down."

Yet despite the challenges it poses, complexity can also be a source of great opportunity for policy makers and for intervention design. Nonlinearity is an opportunity, since a coincidence of several small events can generate a large systemic effect. Near the right thresholds, even very small interventions can have a big impact on the system, "tipping" it to a new state. Understanding heterogeneity in a system can also create an opportunity because it allows interventions to be very closely targeted for maximum impact. And decentralized dynamics are an advantage if they can be harnessed to allow interventions to disseminate on their own through direct imitation or interaction between actors in the system. Tools developed for the study of complex systems can help uncover their underlying dynamics, identifying which areas will be most amenable to policy intervention and where leverage may best be applied for any particular policy goal.

## Modeling Techniques for Complex Systems

An especially valuable tool, both for scientific study and for policy design, is *modeling*. There are many benefits to constructing explicit models, or representations, of a system's behavior (63). Unlike implicit ("mental") models, assumptions in an explicit model are easily testable and boundary conditions can be identified, making them a more reliable basis for decision making. Models can help reduce the list of plausible hypotheses about a system, ruling out those that produce dynamics very different from the real behavior of the system (64). They can lead to surprising or counterintuitive insights about the underlying mechanisms at work in a system and can help focus empirical inquiry by identifying the most critical gaps in data. Finally, models can help test the potential effect of interventions and identify the points of maximum leverage in the system.

A wide array of techniques for modeling exist and are used for many different purposes in many different fields. The complexity of obesity means that the most suitable modeling techniques will have several particular characteristics. First, because of the great breadth in scale of the obesity epidemic, models of obesity may provide the most insight if they capture *multiple levels* of analysis (14,59,60). Modeling a single level at a time does not permit examination of linkages and feedback between levels, nor determination of the relative degree of influence of each level on obesity outcomes. Second, in order to fully capture the dynamics of a complex system, models must also be able to incorporate individual heterogeneity and adaptation over time (see above). Finally, there is a great need for models that provide not just better understanding of the problem and the mechanisms behind it, but that aid design of new and better interventions to slow and reverse the epidemic (65). Therefore, the most useful models will be those that can serve as policy "laboratories," modeling the potential effect of interventions on system dynamics.

Given these criteria, a set of particularly promising tools can be found in computational and simulation techniques drawn from complexity science.

### Agent-based computational modeling

One methodology often used to study complex systems is *agent-based computational modeling* (ABM), a powerful and relatively new approach made feasible by advances in computing. In ABM, complex dynamics are modeled by constructing "artificial societies" on computers. Every individual actor (or "agent") in the system is explicitly represented in computer code. The agents are placed in a spatial context, with specified initial conditions, and given a set of adaptive rules that govern their interaction with each other and with their environment. The interactions and decision processes of the agents produce output at both the individual and aggregate system levels. In this way, the computer simulation "grows" macro-level patterns and trends from the bottom up (64), making it especially well-suited for the study of complex systems. The macro-level patterns generated by the model (eg, changes in distribution of body mass index [BMI], eating patterns, health outcomes) can be directly compared with data to calibrate the model.

Agent-based models maintain a high degree of analytical rigor but offer several particular advantages for modeling complex systems (such as the obesity epidemic). First, ABM allows for substantial diversity among agents — because every individual is explicitly modeled, no "representative agents," compartments, homogeneous pools, or other forms of aggregation are required. Thus agent-based models can easily incorporate both diversity in the types of actors in a system (doctors, patients, insurers) and heterogeneity within each type (in sociodemographics, physiology, genetics, networks, location, psychology, etc). Taking such diversity into account is often critical in designing successful interventions into complex systems.

The agent-based approach also allows great flexibility in cognitive assumptions about individual decision making and information processing. Agents in simulation models need not be "optimizing" but can be simply goal oriented in the context of limited and changing information. This kind of "bounded rationality" is often more plausible for modeling real-world decision making (66) and is an important source of diversity as well.

In addition, agent-based models can incorporate complex feedback dynamics and explicit spatial contexts. At the individual level, they can model multiple interdependent sources of influence on health outcomes. At the aggregate level, they can model the interaction of actors and environments across multiple levels of analysis, as agents can be implemented at multiple levels of scale simultaneously. Agent-based models can include explicit representations of geography from geographic information systems data (67), as well as detailed social network structures and complex neighborhood effects (68). Such representations of space are often difficult to incorporate into standard analytic approaches (69).

A particular advantage of the ABM approach for studying CASs is its focus on mechanisms and ability to study nonequilibrium dynamics. Since complex systems are rarely in equilibrium and are often susceptible to "tipping," this flexibility is especially important. Because of its focus on mechanisms, ABM allows adaptation (evolution, learning, imitation) to be modeled explicitly and drivers of "emergent" social level phenomena to be uncovered. Because it is rule-based, agent modeling has also proven particularly transparent, facilitating research in cross-disciplinary teams (of particular interest for the study of obesity, as argued above) (67,70).

Finally, agent-based models are especially useful as computational laboratories" for policy. With an agent-based model, researchers can systematically explore the potentially complex impacts of each item on the existing menu of policy interventions — and can even uncover novel ones. Agent-based models have been used to study a wide variety of topics in social science and public health (67,68,71-81) and have been able to provide important guidance to policy making and intervention design in several instances (80,81).

An ABM approach to obesity would permit modeling of multiple mechanisms simultaneously, across several levels of scale, with inclusion of important sources of diversity. For example, one set of "agents" might be individual consumers, placed in an environment with opportunities for eating and for physical activity. Within each of these agents could be contained a representation of metabolic mechanisms, genetics, or neurobiology, with the appropriate degree of population diversity reflected in variation between agents. These agents could be embedded in a social and environmental structure with multiple sources of influence from other agent types — peers, parents, media, prices set by markets, etc. Individual behaviors would then adapt over time through interaction with "above the skin" environment and society and would simultaneously be shaped by "below the skin" genetics, metabolism, and neurobiology. This would allow interaction of many different levels and mechanisms (82). Such an approach could offer deeper understanding of the full complexity of the obesity epidemic and permit experimentation with different forms of policy intervention.

### Other simulation methods

Several other powerful computational and simulation techniques are widely used for studying complex systems. System Dynamics (83-86) is a technique in which a system is modeled using 3 core components: stocks (key variables, like population BMI, that increase or decrease over time), flows (rates of change in a stock), and feedback loops (which can connect stocks and flows over time). Once a diagrammatic model of the system is developed, implemented in computer software, and filled in with data for each of the relationships between variables, the dynamics of the system can be explored under many scenarios and sets of assumptions. This technique is particularly effective at giving insight into large systems for which good data exist for most relationships between aggregate variables and makes sensitivity analysis very straightforward. Commercial software for system dynamic modeling is also quite standardized and well-documented. Because it is a more "top-down" approach, however, and key objects are generally represented at a macro level, it does not easily give insight into the individual actor level or incorporate extensive agent heterogeneity, adaptation, or emergence.

Two other common techniques are Dynamic Microsimulation and Markov modeling. Commonly used in economics, Dynamic Microsimulation (87,88) is similar to ABM in its bottom-up, individual-level focus. Although there are many variants of this approach, microsimulations generally assume no interaction *between* components (agents). This simplifies analysis but also makes mechanisms such as social influence, imitation, and contagion difficult to capture. Markov modeling (89,90) also shares several features with ABM, such as its ability to capture distributions of attributes and model their dynamics. In a Markov model, a population of individuals may be in one of a fixed number of "states," with transition probabilities governing movement from one state to the next. Like System Dynamics, and Microsimulation, Markov models are straightforward to implement in standard software packages. However, they are less flexible than agent-based models because they generally assume low dimensional state space and cannot easily handle complex state transition dynamics like path dependence and individual learning.

## Data Requirements for Models

Models advance scientific understanding most effectively and provide the best basis for intervention design and policy making when they are empirically sound. Empirical data can help make model input assumptions as valid as possible and can be used to test the output of models and their power to explain real-world phenomena of interest. Simulation models, of all types described above, are capable of producing large amounts of clean output data; there is rarely as much empirical data available for comparison. A variety of methodologies have been developed for the productive use of both real and simulated data in modeling. Both microlevel data (about individual decision making and biological substrates) and macro-level data (about distributions or flows) can inform the assumptions that go into a model. In cases where few data are available on which to base an individual assumption, comparison of model output with empirical data can be used to calibrate the model. Models can gain greater corroboration when they can "reconstruct" or explain historical cases of particular interest. And simulated data on their own can be used to perform internal consistency checks on model dynamics. For further discussion of these and other strategies for relating data to models, see references 57,67,91, and 93. It is important to note that models can also be very useful even where few data are available — such models can help to build theory, generate hypotheses, and identify mechanisms capable of generating key "stylized facts" of interest (58,71,73-75,94,95).

## Application and Future Directions

The most effective models of the obesity epidemic are likely to be those that can capture multiple mechanisms at multiple levels, integrate micro and macro data and dynamics, account for significant heterogeneities, and allow for policy experimentation. The computational and simulation modeling techniques discussed in the previous section have great potential to meet all of these goals. Their application to the obesity epidemic will be challenging — there will be important data that are not yet available, uncertainty about many assumptions, and many key mechanisms whose inner workings remain unknown. But modeling can still be quite effective. Indeed, modeling and empirical study are often most productive when they work together iteratively. Modeling can help direct empirical inquiry by highlighting the most significant and critical gaps in data and by generating theory and hypotheses for testing. And new data and evidence can inform a revised and updated set of models.

One promising strategy for modeling the obesity epidemic may be to make use of a *modular* approach. Although the system driving the obesity epidemic is complex, the best models are parsimonious. Modeling all the levels of the system simultaneously from the outset can make assessment of the relative contributions at each level difficult and complicate efforts to understand the mechanisms at work in any specific level. A modular approach, by contrast, would allow separate consideration of each level of analysis while still permitting straightforward integration to study multilevel feedbacks and interactions. Such an approach would begin with a separate "module" (model) for each level of analysis (eg, genetics, brain, family, social networks, built environment), incorporating the best available theory regarding mechanisms and pathways of effect. The modules would share a common empirical "testbed" of outcome data, allowing testing of the ability of each to explain variance in outcomes. Various combinations of modules could then be explored and tested against the same outcome data, building slowly towards a model covering the full breadth of the system by integrating all of the modules. A similar scaling strategy could be used for the scope of the "testbed." Initially, the models might be applied to outcomes in a small, relatively homogeneous population. Later, the scope could be expanded to a much larger, more diverse population.

This type of modular modeling has been highly successful in fields such as engineering and ecology (96-98) but has not yet been applied using the computational techniques discussed here in a public health context.

## Conclusion

Several attributes of the obesity epidemic — the great breadth in levels of scale involved, the substantial diversity of relevant actors, and the multiplicity of mechanisms implicated — make it an especially challenging problem. This article has shown that these attributes are characteristic of a CAS and argued that obesity is indeed driven by such a system. Computational and simulation modeling techniques drawn from the fields of complexity and systems science represent an especially promising avenue for future study of the rich and complex dynamics of obesity and for the design of effective interventions and policies to combat it.
